# The genetic variant *rs55986091* HLA-DQB1 is associated with a protective effect against cervical cancer

**DOI:** 10.3389/fonc.2023.1207935

**Published:** 2023-08-08

**Authors:** Michael A. Vinokurov, Konstantin O. Mironov, Elvira A. Domonova, Tatiana N. Romanyuk, Anna A. Popova, Vasiliy G. Akimkin

**Affiliations:** Central Research Institute of Epidemiology of the Federal Service for Surveillance on Consumer Rights Protection and Human Wellbeing, Moscow, Russia

**Keywords:** high-risk oncogenic human papillomavirus, single nucleotide polymorphism, cervical cancer, genetic predisposition, personalized medicine

## Abstract

**Introduction:**

Cervical cancer (CC) is a prevalent malignancy affecting women globally. The primary causative factor of CC is the high-risk oncogenic human papillomavirus (HR-HPV). However, it is noteworthy that not all women infected with HR-HPV develop cancer, indicating the potential involvement of genetic predisposition in the development of CC. This study aims to identify genetic risks and their distribution in groups of women with different epidemiological features of HR-HPV.

**Materials and methods:**

A comparison was conducted among four groups of women, comprising 218 HPV-negative women, 120 HPV-positive women, 191 women diagnosed with cervical intraepithelial neoplasia (CIN) grade 2 or 3, and 124 women diagnosed with CC. The analysis focused on four single nucleotide polymorphisms (SNPs): *rs55986091* in *HLA-DQB1*, *rs138446575* in *TTC34*, *rs1048943* in *CYP1A1*, and *rs2910164* in *miRNA-146a*.

**Results:**

The rs55986091-A allele exhibited a protective effect within the “CC” group when compared to the “HPV-Negative” group (OR = 0.4, 95% CI= 0.25-0.65) using a log-additive model. Additionally, similar protective effects were observed in the “CIN 2/3” group compared to the “HPV-Negative” group (OR = 0.47, 95% CI = 0.28-0.79).

**Conclusion:**

The data obtained emphasize the importance of developing PCR-based diagnostic kits for the identification of SNP alleles, particularly for rs55986091, among HR-HPV-positive women within the Russian population.

## 1 Background

Cervical cancer (CC) is globally recognized as the fourth most common and fatal oncological disease among women. In 2020, approximately 604,000 new cases of CC were reported, resulting in over 340,000 fatalities and contributing to 7.7% of all cancer-related deaths (1). The incidence of CC in Russia has witnessed a concerning rise over the past decade (2009 to 2019), with a nearly 12-13% increase (from 14,000 to 17,000 cases and from 112 to 127 cases per 100,000 population). This escalating trend highlights the societal significance of this disease. Notably, women aged 30 to 44 years who are in their socially active and reproductive years account for 32.4% of CC cases (2).

According to Russian cancer statistics from 2019, the existing preventive measures for cervical precancerous conditions have demonstrated limited effectiveness, particularly concerning vaccination programs. Furthermore, there is a lack of awareness among the population regarding the significance and accessibility of CC screening and a lack of motivation for undergoing preventive examinations. Consequently, the mortality rate for CC remains largely unchanged (2).

Infection with high-risk oncogenic human papillomavirus (HR-HPV) has been established as a confirmed carcinogenic factor for CC. Within this study, 14 HPV types were identified as highly carcinogenic, namely, types 16, 18, 31, 33, 35, 39, 45, 51, 52, 56, 58, 59, 66, and 68 (3). The association between HR-HPV and CC has been found to be stronger than the association between smoking and lung cancer (4). Interestingly, among women who test positive for HPV, the incidence of CC is only 0.015%, suggesting that genetic susceptibility may play a role (5).

It is worth noting that HPV has the potential to induce the development of various tumors, not only in women but also in men. These include anogenital lesions, head and neck tumors, and other types of tumors. As a result, the social and economic burden associated with HPV-associated diseases may have been significantly underestimated (6).

The identification of single nucleotide polymorphisms (SNP) alleles associated with diseases enables the detection of potential inherited predispositions to the development of pathological conditions during the asymptomatic phase. This aids in the timely implementation of diagnostic and treatment measures (7).

Given the lengthy asymptomatic period, sexual transmission, and the impact on women of reproductive age, assessing the genetic risk of developing CC holds significant clinical importance. This is particularly relevant for high-risk groups, including women infected with HIV (8).

In our previous study, we conducted an analysis of SNPs associated with CC risk derived from genome-wide association studies (GWAS) and case-control studies, including a meta-analysis (9). For this particular publication, we focused on examining four specific SNPs: *rs55986091* in *HLA-DQB1*, *rs138446575* in *TTC34*, *rs1048943* in *CYP1A1*, and *rs2910164* in *miRNA-146a*.

Previously, the *rs55986091* SNP (with the protective allele A) was examined in a single GWAS (10). The study revealed a significant association, with an OR of 0.66, a 95% CI of 0.60-0.72, and a p-value of less than 1×10^-11^, specifically for the group with cervical intraepithelial neoplasia grade 3 (CIN3) or invasive CC (10). Many reviews acknowledge the significance of rs55986091 as the SNP most strongly associated with CC, referring to GWAS findings (11, 12).

The rs138446575 SNP (with the risk allele T) was also examined in the same GWAS study (10). The findings revealed a significant association, with an OR of 2.39 and a 95% CI of 1.75-3.27, at a p-value of 4.97×10^−8^, specifically within the groups diagnosed with invasive CC. The *rs138446575* SNP is located between the *TTC34* and *PRDM16* genes in a region whose functions have not yet been fully identified.

The *rs1048943* SNP (with the risk allele C) has been investigated in meta-analyses, revealing significant associations with CC. Among women from India, the meta-analysis showed an OR of 2.34, with a 95% CI of 1.37-3.99 (13). For other populations, the analysis demonstrated an OR of 2, with a 95% CI of 1.33-3 (14). In the analysis conducted on the European population (white population of Europe and the United States), a positive trend in CC development was observed, with an OR of 2.22 and a 95% CI of 1.48-3.33 (9). The *rs1048943* SNP causes an amino acid substitution in the *CYP1A1* gene, resulting in reduced enzymatic activity and being associated with the development of other types of cancers (14, 15).

The *rs2910164* (with the risk allele C) has been examined in previous articles (16, 17), and statistically significant associations have been identified. In one study, the analysis revealed an OR of 1.35, with a 95% CI of 1.06-1.74 (16). Another study reported an OR of 1.41, with a 95% CI of 1.17-1.70 (17). These significant associations were observed specifically within the Asian population. Additionally, the *rs2910164* has been included in the assessment of risks for the European population.

The primary objective of this study was to determine the genetic risks and their distribution among different groups of women with varying HR-HPV clinical presentations.

## 2 Materials and methods

### 2.1 Oligonucleotide design

In this study, a real-time PCR-based approach was performed for the discrimination of SNP alleles (18). The design of the probes used in this approach was based on the methodology employed in the study (19). Primers were selected for pyrosequencing of all SNPs, and an additional Sanger sequencing was chosen specifically for rs55986091. The specific sequences of the oligonucleotides used in the study can be found in **Table 1**.

**Table 1:** Oligonucleotide sequences.

SNP	Name (concentration, µM)	5’–3’ sequence
rs138446575	6575-F (0.4)	CACCTGGCAACTTGCAGACAG
	6575-R (0.4)	biotin-AGGACGTCCTCGCCACAT
	6575-S (0.3)	GGTGCCGCCAGCCTT
	6575-C (0.04)	(FAM)G+CC+TT+**C**CTG+GGT (BHQ1)
	6575-T (0.12)	(R6G)AG+CC+TT+**T**C+TGG+GT(BHQ1)
rs55986091	6091-F (0.6)	TCTTTTTTATTTTTCCTAAGAGTCGAT
	6091-R (0.6)	biotin-GCAATTTTAATTTCGCCTCAGTC
	6091-S (0.3)	GCATCTCATACACCACCATGCAC
	6091-S2 (0.6)	CAAAAACCAGCAAGTTTTTATTAG
	6091-G (0.12)	(FAM)T+TA+TT+AG+**G**GA+T+TT+TCA (BHQ1)
	6091-A (0.12)	(R6G)T+T+AT+TAG+**A**G+A+TT+T+TCAA (BHQ1)
rs1048943	8943-F (0.4)	CGACAAGGTGTTAAGTGAGAAGGTG
	8943-R (0.4)	biotin-AGGATAGCCAGGAAGAGAAAGACC
	8943-S (0.3)	GGAAGTGTATCGGTGAGACC
	8943-A (0.08)	(FAM)GA+GACC+**A**T+TGC+CC (BHQ1)
	8943-G (0.12)	(R6G)G+AG+ACC+**G**+TT+GCC (BHQ1)
rs2910164	10164-F (0.4)	CTGAATTCCATGGGTTGTGTCAGT
	10164-R (0.4)	biotin-GATGACAGAGATATCCCAGCTGAA
	10164-S (0.3)	GGTTGTGTCAGTGTCAGACCT
	10164-G (0.04)	(FAM)C+AG+ACCT+**G**TG+A+AA+TT(BHQ1)
	10164-C (0.12)	(R6G)C+AG+ACCT+**C**TG+A+AA+TT(BHQ1)

FAM, R6G – fluorophores; BHQ1 – a quencher. The LNA (locked nucleic acid) is indicated as “+_”. F, a forward primer; R, a reverse primer; S, a primer for pyrosequencing; S2, a primer for Sanger sequencing. The polymorphic nucleotides are highlighted in bold.

### 2.2 DNA extraction, PCR, and sequencing

DNA extraction from whole blood samples and cervical scrapings (exocervix and endocervix) collected in the BD SurePath transport medium (BD Diagnostics, USA) was performed using the RIBO-prep (Registration Certificate FSR 2008/03147 of 15/9/2008) and AmpliSens^®^ DNA-sorb-D (Registration Certificate RZN 2015/3503 of 27/3/2019) kits. The investigation of HPV types was conducted using the AmpliSens^®^ HR HPV screen-titre-14-FL (Registration Certificate RZN 2017/5387 of 22/2/2019) and AmpliSens^®^ HR HPV geno-type-titre-FL (Registration Certificate RZN 2017/6533 of 27/3/2019) kits. These kits enabled HR-HPV genotyping for 14 specific types, namely, 16, 18, 31, 33, 35, 39, 45, 51, 52, 56, 58, 59, 66, and 68.

The PCR reactions were performed using a reaction mix of 25 μl, which included primers, probes, and dNTP (0.44 mM) in a volume of 10 μl. Additionally, 0.5 μl of TaqF polymerase and 4.5 μl of RT-PCR-mix-2 FEP/FRT reagents were added. The extracted DNA (10 μl) was also included in the reaction mix. Real-time PCR was carried out using the Rotor-Gene Q cycler (Qiagen, Germany) and DTprime (DNA-Technology, Russia) instruments. The following cycling conditions were employed: an initial denaturation step at 95°C for 15 minutes (1 cycle), followed by 45 cycles of denaturation at 95°C for 5 seconds, annealing at 60°C for 20 seconds, and extension at 72°C for 10 seconds. During the amplification process, a fluorescent signal was detected at 60°C. The amplification results were analyzed by setting the threshold at 10% of the highest value of the fluorescent signal for each channel. All the reagents and kits used for PCR and DNA extraction were manufactured by AmpliSens, Russia, and provided by the Central Research Institute of Epidemiology of Rospotrebnadzor.

The PCR products were sequenced using the PyroMark Q24 (Qiagen, Germany) and 3500xL (Applied Biosystems, United States) instruments, and reagent kits were recommended by the equipment manufacturers.

### 2.3 Description of the sample groups

A retrospective study was conducted on biological samples collected between 2017 and 2021 at the Central Institute of Epidemiology. Informed consent was obtained from all patients regarding the use of their DNA samples, and samples without informed consent were excluded from the study. The collected samples were categorized into five distinct groups:

The “Population Control” group consisted of randomly selected whole-blood samples obtained from donors whose sex, age, and HPV infection status were unknown.

The “HPV-Negative” group included women who tested negative for high-risk human papillomavirus (HR-HPV) and had negative results for intraepithelial lesion of malignancy (NILM) during cervical screening over a 5-year monitoring period. The cervical scrapings were used for this assessment.

The “HPV-Positive” group consisted of women who tested positive for high-risk human papillomavirus (HR-HPV) and had negative results for intraepithelial lesion of malignancy (NILM) during cervical screening over a 5-year monitoring period. Within this group, 29% of women showed elimination of the virus, while 71% demonstrated persistent HR-HPV infection.

The “CIN 2/3” group consisted of women who tested positive for high-risk human papillomavirus (HR-HPV) and had cytologically and/or histologically confirmed CIN grade 2 or 3.

The “CC” group comprised women who tested positive for high-risk human papillomavirus (HR-HPV) and had histologically confirmed carcinoma *in situ* or squamous cell carcinoma of the cervix.

Cytological and histological evaluations, as well as the diagnosis of carcinoma, were conducted based on investigations of cervical scrapings. All the samples included in the study were collected from residents of Moscow, Russia. Samples from the “HPV-Negative,” “HPV-Positive,” “CIN 2/3,” and “CC” groups were recruited between 2017 and 2021. Samples from the “Population Control” group were collected between 2018 and 2020.

Detailed information about the samples is given in **Table 2**.

**Table 2:** Description of the groups.

Groups	Sample size	Age Median (IQR*)	Types of HPV detected
Population Control	137	–	–
HPV-Negative	218	23-66 45 (36-53)	–
HPV-Positive	120	21-70 36 (30-38)	16,18,31,33, 35, 39, 45, 51, 52, 56, 58, 59, 66, 68
CIN 2/3	191	19-73 35 (31-40)	16,18,31,33, 35, 39, 45, 51, 52, 56, 58, 59, 66, 68
CC	124	16-75 36 (33-45)	16,18,31,33, 35, 39, 45, 51, 52, 56, 58, 59, 68

*Interquartile range.

### 2.4 Statistical data

The obtained allele data were compared with the allele frequencies from the Ensembl database for Europeans and validated using the Hardy-Weinberg equilibrium and Pearson’s χ2 test. The associations of the SNPs were assessed using logistic regression and expressed as OR with 95% CI for standard genetic models in case-control studies (20). The Akaike information criterion was calculated to determine the most preferred model. The statistical analysis of the results was performed using the R program (21). The linkage disequilibrium analysis, which examines the non-random association of alleles at different loci, was conducted using LDMatrix from the National Cancer Institute (22). To account for multiple testing, the p-values were adjusted using the Bonferroni correction by multiplying the obtained p-values by the number of SNPs and comparisons.

## 3 Results

The results of genotyping of the samples are given in **Table 3**.

**Table 3:** The results of genotyping of the groups.

Group	rs2910164 *miRNA-146a*			rs138446575 *TTC34*			rs1048943 *CYP1A1*			rs55986091*HLA-DQB1*		
	GG	GC	CC	CC	CT	TT	TT	TC	CC	GG	GA	AA
**Population control, n (%)**	82 (60)	49 (36)	6(4)	132 (96)	5(4)	0	127 (93)	10 (7)	0	112(82)	7(5)	18 (13)
**HPV-negative, n (%)**	135 (62)	74 (34)	9(4)	208 (95)	10 (5)	0	202 (92.5)	15 (7)	1 (0.5)	165 (76)	16 (7)	37 (17)
**HPV-positive, n (%)**	78 (65)	36 (30)	6(5)	116 (97)	4(3)	0	112 (93)	7(6)	1(1)	101 (84)	9(8)	10(8)
**CIN 2/3, n (%)**	116 (61)	64 (34)	11 (5)	183 (96)	8(4)	0	174 (91)	15 (8)	2(1)	166 (87)	7(4)	18(9)
**CC, n (%)**	82 (66)	36 (29)	6(5)	120 (97)	4(3)	0	114 (92)	9(7)	1(1)	115 (93)	5(4)	4(3)

In the initial stage of the study, we conducted genotyping of the Population Control group for four markers and compared the allele frequencies with the data obtained from the Ensembl database (23). To determine if there were any significant differences in allele frequencies between the European and Russian populations, a χ2 test was performed. The analysis revealed that the p-value obtained from the χ2 test was greater than 0.2, indicating that no statistically significant differences were observed in the allele frequencies between the European and Russian populations for these markers.

Based on the analysis, no significant differences were found for the markers rs2910164, *rs138446575*, and *rs1048943* between the groups. However, significant differences were observed for the marker *rs55986091* in *HLA-DQB1* across all four inheritance models. The codominant and recessive models specifically showed the lowest OR of 0.16. The calculation of OR for the five genetic models can be found in **Table 4**.

**Table 4:** Comparison of the «HPV-Negative» and «CC» groups by *rs55986091 HLA-DQB1*.

Model of Inheritance	Genotype	CC	HPV-Negative	OR (95% CI)	p-value	AIC*	BIC**
Codominant	GG	115	165	1		436	448
	GA	5	16	0.45 (0.16-1.26)	>0.5		
	AA	4	37	0.16 (0.05-0.45)	<0.005		
Dominant	GG	115	165	0.24 (0.12-0.51)	<0.005	436	444
	GA/AA	9	53				
Recessive	GG/GA	120	181	0.16 (0.06-0.47)	<0.005	437	445
	AA	4	37				
Overdominant	GG/AA	119	202	0.53 (0.19-1.48)	>0.5	452	460
	GA	5	16				
Log-additive	–			0,4 (0,25-0,65)	<0.005	434	442

* – Akaike information criterion (AIC).

** – Bayesian information criterion (BIC).

The analysis of the AICs and BICs indicated that the log-additive model was the most likely inheritance model for the rs55986091 genetic variant. This suggests that carrying the A allele may provide a protective effect against CC development. On the other hand, the G allele was found to increase the risk of CC (OR=2.49, 95% CI=1.55-4.01).

Specifically, in the Dominant model, individuals carrying the GG and GA genotypes had an increased risk of CC development with an OR of 6.13 (95% CI=2.13-17.65). In the “CC” group, there were 120 individuals with the GG and GA genotypes compared to 4 individuals with the AA genotype. In the “HPV-Negative” group, there were 181 individuals with the GG and GA genotypes compared to 37 individuals with the AA genotype. These findings suggest a significant association between the GG and GA genotypes and an increased risk of CC.

The frequencies of observed genotypes deviated from the Hardy-Weinberg equilibrium (p-value<0.0001).

In order to compare the HPV-Negative and CIN 2/3 groups, we examined four genetic markers. Among these markers, *rs55986091* showed a consistent association, with the dominant model having the lowest OR of 0.47. After adjusting for multiple comparisons using the Bonferroni correction, the statistically significant differences remained significant for the dominant and log-additive genetic models.

For further details and specific OR values, refer to **Table 5**.

**Table 5:** «HPV-Negative» and «CIN 2/3» by *rs5598609 HLA-DQB1*.

Model of inheritance	Genotype	CIN 2/3	HPV-Negative	OR (95% CI)	p-value	AIC	BIC
Codominant	GG	166	165	1	–	441	452
	GA	7	16	0.44 (0.18-1.09)	>0.5		
	AA	18	37	0.49 (0.27-0.89)	>0.1		
Dominant	GG	166	165	0.47 (0.28-0.79)	<0.05	440	448
	GA/AA	25	53				
Recessive	GG/GA	173	181	0.51 (0.28-0.93)	>0.05	439	446
	AA	18	37				
Overdominant	GG/AA	184	202	0.48 (0.19-1.20)	>0.5	444	451
	GA	7	16				
Log-additive	–			0.67 (0.5-0.9)	<0.05	439	447

The analysis using the Akaike information criterion (AIC) and Bayesian information criterion (BIC) reaffirmed that the log-additive inheritance model is the most likely model for this particular locus.

Furthermore, we compared the HPV-Negative and HPV-Positive groups using four genetic markers. While some statistical differences were observed in the recessive model for rs55986091, these differences were considered insignificant after applying the Bonferroni correction to account for multiple comparisons. For more comprehensive information, please refer to **Table 6**.

**Table 6:** «HPV-Negative» and «HPV-Positive» by *rs55986091 HLA-DQB1*.

Model of inheritance	Genotype	HPV-Positive	HPV-Negative	OR (95% CI)	p-value	AIC	BIC
Codominant	GG	101	165	1	–	441	452
	GA	9	16	0.92 (0.39-2.16)	>0.5		
	AA	10	37	0.44 (0.21- 0.93)	>0.1		
Dominant	GG	101	165	0.59 (0.33-1.05)	>0.1	440	448
	GA/AA	19	53				
Recessive	GG/GA	110	181	0.44 (0.21- 0.93)	>0.1	439	446
	AA	10	37				
Overdominant	GG/AA	111	202	1.02 (0.44- 2.39)	>0.5	444	451
	GA	9	16				
Log-additive	–			0.69 (0.49-0.98)	>0.1	439	447

**Figure 1:**
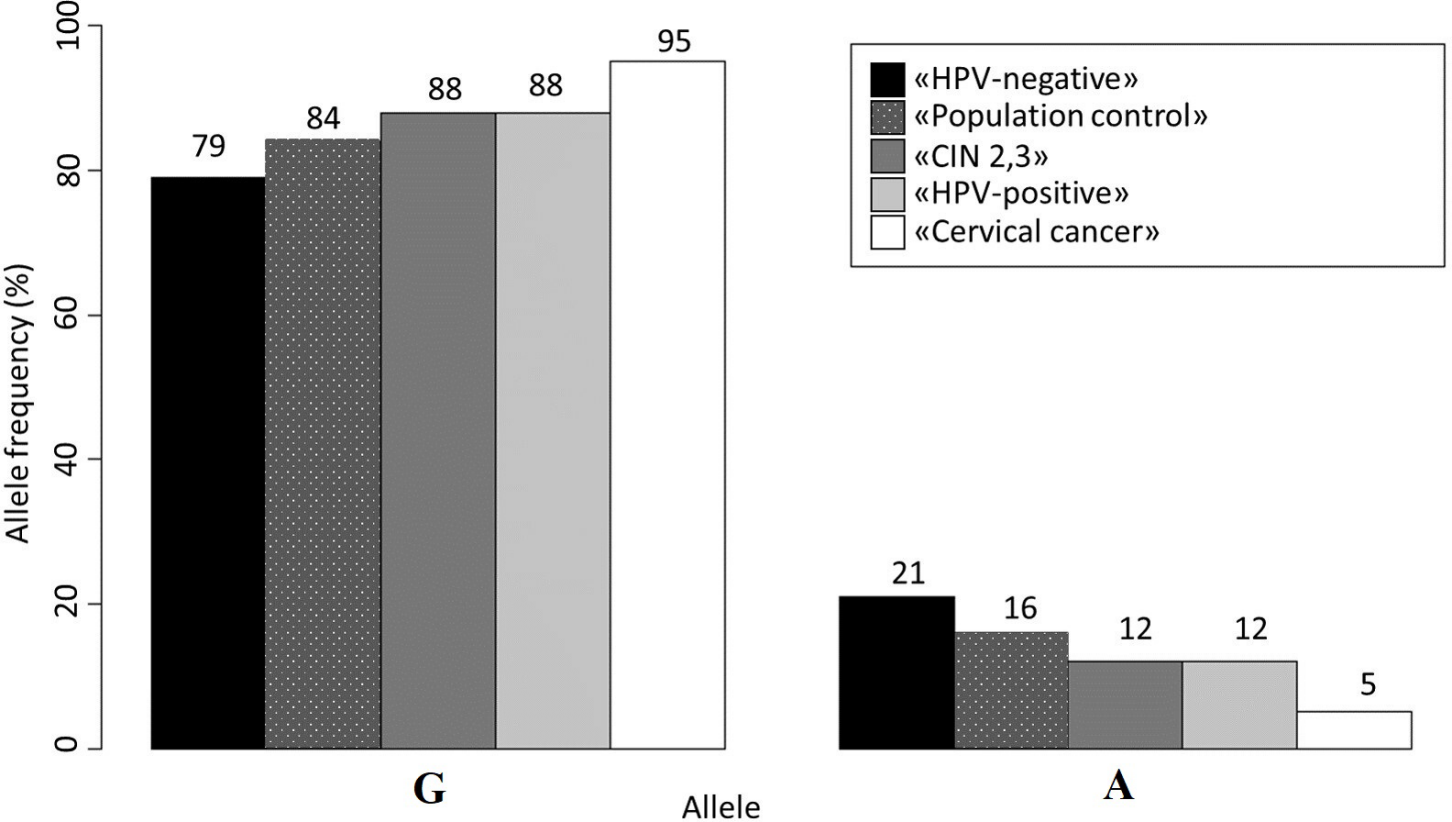
Allele frequencies for *rs55986091* in all studied groups.

The analysis of allele frequencies revealed that the “Population Control” group had a slightly higher frequency of the A allele compared to women without HPV, but this difference was not statistically significant (p-value>0.05). However, the frequency of the A allele in women with CIN 2/3 was similar to that in the “HPV-positive” group and significantly different from the “HPV-Negative” group (p-value<0.05). For a more detailed overview of the allele frequencies, please refer to **Figure 1**.

The dominant HPV types in the HPV-infected group were 68, 16, and 31 (in 14%, 13%, and 13% of people, respectively). In the CIN 2/3 group, 16, 31, and 33 HPV types were most often detected (in 53, 19, and 17% of people, respectively). In the group of women with CC, the HPV types often identified were 16, 18, and 33 (in 53, 13, and 8% of people, respectively). Detailed information about the percentage of people infected with different types of HPV in three groups is provided in **Supplementary Table S1**. The association of HPV type and risk genotype in the three groups was also considered. However, no statistically significant differences were found in the distribution of HPV types depending on the genotype in each of the groups.

## 4 Discussion

The rs55986091-A allele exhibited a protective effect in the “HPV-Negative” group compared to the “CC” group. The rs55986091 is located in the regulatory region between the HLA-DQA1 and HLA-DQB1 genes (23). In particular, this sequence functions as a super-enhancer for the HLA-DQB1 gene, which is responsible for the transcription and subsequent differentiation of cells (24). The HLA-DQB1 gene is part of the human leukocyte antigen (HLA) complex class II, which plays a crucial role in presenting antigens from external sources to the immune system (25). The upregulation of HLA class II expression and the high concentration of antigen presented by HLA class II molecules on antigen-presenting cells trigger the activation of CD4+ T cells, which is vital for mounting an immune response against pathogens (26).

We propose two hypotheses regarding the influence of this SNP on the development of CC in women:

1. There is a genetic linkage between the observed SNP and a specific allele of the human leukocyte antigen (HLA) gene. This HLA allele is believed to enhance the effectiveness and/or prolong the duration of the immune response in individuals who have received the HPV vaccine.
2. This SNP affects the expression of the HLA-DQB1 gene, whose product may be associated with a protective effect against cancer.

The first hypothesis is supported by several pieces of evidence. Previous studies have demonstrated the protective effects of certain HLA alleles, such as HLA-DQB1*02, HLA-DQB1*0603, DQB1*03, DQB1*0604, DQB1*0501, and DQB1*0603, in CC and other precancerous lesions (27). Carriers of these HLA alleles may exhibit an enhanced immune response after receiving the HPV vaccine, which could contribute to a lower risk of CC development. Similar findings have been observed in the context of hepatitis B, where DQB1*06 variants were associated with a more durable immune response in individuals who received the hepatitis vaccine (28).

One limitation of the present study is the lack of information regarding HPV vaccination in the study group. Adjusting for vaccination status could provide a better understanding of the observed effects. In future research, this limitation should be addressed by incorporating data on HPV vaccination and employing bioinformatics tools to investigate the linkage disequilibrium of this SNP with HLA alleles DQB1 and DQA1.

The second hypothesis also has a basis in the available evidence and should be further investigated in upcoming studies. Given that this SNP is located in the regulatory region *of HLA-DQB1*, it is possible that it is associated with alterations in gene expression. Reduced or impaired expression of HLA molecules has been linked to the development of CIN and CC (27). Previous studies have also observed associations between polymorphic variants and HLA alleles, such as the linkage disequilibrium between rs2395029 and HLA-B*57:01, which are 100 kb apart (29). Although the connection of CC has been established specifically for rs55986091, it is noteworthy that there are more than 10 other SNPs (R2>0.5) (22) associated with rs55986091 within the super-enhancer region (NC_000006.12:c32651217-32660516). This suggests that variations in the nucleotide sequence spanning over 9,000 bases, particularly those involving the A allele in rs55986091, may have a higher affinity for binding RNA polymerase. Consequently, the immune response triggered in the presence of this allele might be more potent, not only in individuals infected with HPV but also in those affected by other infections (29, 30). These findings are further supported by our data indicating statistical differences in allele frequencies between HPV-negative women and women who were infected and subsequently cleared the virus.

In conclusion, the data indicate an association between this SNP and the immune response to HPV entry into the cell. However, further research is required on the deeper mechanisms underlying this.

Significant deviation from the Hardy-Weinberg equilibrium was observed for rs55986091 (p-value<0.001), indicating a departure from the expected genotype frequencies. Specifically, the frequency of observed heterozygotes was lower than expected, while the frequency of observed homozygotes in both genotypes was slightly higher than expected. Further analysis of the super-enhancer region (NC_000006.12:c32651217-32660516) revealed the presence of at least 100 SNPs with similar genotype distributions.

Based on the findings, it is plausible to suggest that homozygous SNP variants in this region exhibit a higher affinity for recognition by RNA polymerase, resulting in enhanced gene expression and a more robust immune response. Additionally, there may be a genetic linkage between rs55986091 and one of the SNPs situated within the exon, influencing the encoding of the peptide allele (31).

Graffelman et al. previously observed a deficiency of heterozygotes between genes as well as an excess within the genes for HLA class II, which they attributed to the presence of null alleles and sequence errors (32). However, in the case of our SNP, this explanation is not applicable as we did not find any differences between our sample and the Ensembl database. We also conducted pyrosequencing and Sanger sequencing to validate our data. Moreover, the disequilibrium observed persisted across all samples and populations in the Ensembl database. Therefore, we can rule out sequence errors and instead consider explanations such as natural selection or non-random mating as potential factors contributing to the observed disequilibrium. In future studies, we plan to further investigate this aspect by examining genotypes based on the aforementioned SNP in individuals with other infections, as well as measuring the gene expression levels associated with specific genotypes of this SNP.

Additionally, the association of the HPV type with one of the host genotype variants was considered. We did not find a statistically significant association for any of the types in each group. We assume that with an increase in samples, it will become more realistic to establish a relationship between this SNP and the type of HPV. The realization of the carcinogenic potential of the virus is influenced by many factors (smoking, lifestyle, age, and others), including the presence of a genetic predisposition, which is realized by a combination of unfavorable combinations of risk alleles in SNP.

One limitation of this study is the absence of adjustment for demographic factors such as age, smoking status, occupation, vaccination history, and specific HPV types. These factors can potentially influence the development of CC and could have confounding effects on the observed associations. Additionally, the lack of clinical data on patients further limits the comprehensive understanding of the relationships between genetic variants and disease outcomes. Future studies should consider incorporating these demographic factors and clinical data to obtain a more comprehensive analysis.

This study focused exclusively on CC and did not investigate other HPV-associated cancers. Therefore, it is important to replicate the findings in samples of patients with head and neck cancer, including male patients, to determine the association of this SNP with HPV persistence in different cancer types.

The identified associations between specific SNPs and the risk of developing CC have significant implications for the development of screening programs aimed at identifying individuals at higher risk. Additionally, these findings play a crucial role in personalized management approaches for patients infected with high-risk HPV, particularly for individuals with a positive HIV status (8). The personalized approach takes into account individual genetic profiles and can contribute to more effective management and treatment strategies for HPV-associated cancers.

## 5 Conclusion

In the Russian population, the presence of the rs55986091 variant was observed in AA genotype carriers with an OR of 0.16, and in GG and GA genotype carriers with an OR of 6.13. Identifying both individual and population risks of CC development will improve the monitoring of individuals infected with high-risk HPV (HR-HPV). This will increase their motivation for vaccination and cervical examinations, thereby making a significant contribution to reducing CC-related mortality rates.

The findings highlight the importance of developing and conducting clinical trials for an allele discrimination assay targeting rs55986091 in HR-HPV-positive women. Future research plans involve investigating the underlying mechanisms by measuring the expression level of HLA-DQB1 in individuals with different genotype variants. Additionally, testing other SNPs that have shown statistically significant results in previous studies will be pursued (9). The data obtained from these studies will help in the creation of an analytical algorithm based on machine learning for calculating individual relative risks, further improving risk assessment and personalized management strategies for CC.

## Data availability statement

The original contributions presented in the study are included in the article/**Supplementary Material**, further inquiries can be directed to the corresponding author/s.

## Ethics statement

The studies involving human participants were reviewed and approved by The Council on Bioethics of the Central Research Institute of Epidemiology. The patients/participants provided their written informed consent to participate in this study.

## Author contributions

MV: Writing - Original Draft, Formal analysis, Investigation, Visualization. KM: Methodology, Data Curation, Writing - Review and Editing, Investigation. ED: Conceptualization, Methodology, Writing - Review and Editing, Data Curation. TR: Conceptualization, Investigation, Writing - Review and Editing. AP: Conceptualization, Data Curation, Writing - Review and Editing. VA: Supervision, Resources, Project administration, Writing - Review and Editing. All authors contributed to the article and approved the submitted version.
