# Supplementary material for: The genetic variant rs55986091 HLA-DQB1 is associated with a protective effect against cervical cancer
Source: Front Oncol. 2023 Aug 8;13:1207935. doi: 10.3389/fonc.2023.1207935 (PMC10443639; doi:10.3389/fonc.2023.1207935)
Supplement: Supplementary file 1 [file Table_1.docx]

**Supplementary Table S1.** The percentage of people infected with different types of HPV

| HPV type | HPV-positive | CIN 2/3 | CC |
| --- | --- | --- | --- |
|  | n (%) | n (%) | n (%) |
| 16 | 16 (13) | 98 (53) | 67 (53) |
| 18 | 4 (3) | 8 (4) | 16 (13) |
| 31 | 16 (13) | 35 (19) | 6 (5) |
| 33 | 10 (8) | 31 (17) | 10 (8) |
| 35 | 2 (2) | 6 (3) | 6 (5) |
| 39 | 9 (8) | 5 (3) | 2 (2) |
| 45 | 7 (6) | 13 (7) | 12 (9) |
| 51 | 6 (5) | 7 (4) | 1 (1) |
| 52 | 6 (5) | 15 (8) | 4 (3) |
| 56 | 7 (6) | 6 (3) | 2 (2) |
| 58 | 7 (6) | 14 (8) | 4 (3) |
| 59 | 3 (2) | 1 (1) | 2 (2) |
| 66 | 4 (3) | 5 (3) | 1 (1) |
| 68 | 17 (14) | 11 (6) | 3 (2) |
| total | 120 | 191 | 124 |
